# Optimizing planting density for enhancing the synergistic effect of electrokinetic-phytoremediation in petroleum-contaminated soils

**DOI:** 10.3389/fpls.2026.1810883

**Published:** 2026-04-13

**Authors:** Peng Gao, Songyan Liu, Wei Kou, Jiawei Jing

**Affiliations:** 1 College of Engineering, Shenyang Agricultural University, Shenyang, Liaoning, China; 2 College of Chemistry and Environmental Engineering, YingKou Institute of Technology, Yingkou, Liaoning, China; 3 Postdoctoral Station of Agricultural Resources and Environment, College of Land and Environment, Shenyang Agricultural University, Shenyang, Liaoning, China; 4 Key Laboratory of Arable Land Conservation (Northeast China), Ministry of Agriculture and Rural Affairs, Shenyang, Liaoning, China; 5 National Engineering Research Center for Efficient Utilization of Soil and Fertilizer Resources, Shenyang, Liaoning, China

**Keywords:** electrokinetic-phytoremediation, petroleum contamination, planting density, synergistic effect, tall fescue

## Abstract

Electrokinetic-phytoremediation is an environmentally sustainable technology with significant potential for remediating petroleum-contaminated soils. The integration of vegetation to enhance electrokinetic remediation has been extensively studied. However, the impact of planting density on this combined approach remains poorly understood. This study investigates the effect of varying planting densities of tall fescue (*Festuca arundinacea*) on the efficacy of electrokinetic-phytoremediation for petroleum-contaminated soil. Over a 60-day remediation period, the combined electrokinetic and phytoremediation treatments at planting densities of 0.5, 1, 2, and 3 plants/cm^2^ resulted in increased petroleum removal rates of 2.37%, 6.75%, 6.15%, and 5.45%, respectively, compared to electrokinetic remediation alone. Additionally, as planting density increased, soil water-soluble ion concentrations and the degree of compression in the soil colloid double layer thickness progressively decreased during the electrokinetic-phytoremediation process, while soil zeta potential and water-holding capacity initially increased before stabilizing. Planting density had a significant impact on the synergy between the plants and electrokinetics; the synergistic effect initially increased with density but then declined. The optimal planting density for tall fescue in electrokinetic-phytoremediation was found to be 1 plant/cm^2^. This study offers both theoretical insights and empirical data to support the application of electrokinetic-phytoremediation for petroleum-contaminated soil remediation.

## 1 Introduction

Soil contamination with petroleum represents a critical environmental challenge. Petroleum pollutants are highly hazardous to human health and ecosystems due to their carcinogenic, teratogenic, and mutagenic effects (Ambaye et al., 2022). Electrokinetic remediation has proven effective in removing petroleum pollutants from soil. This technology leverages electrokinetic effects and electrochemical oxidation to directly or indirectly remediate contaminated soils (Probstein and Hicks, 1993). The applied electric field facilitates the migration of contaminants and nutrients, thereby enhancing the degradation of pollutants (Cameselle, 2015; Mary M, 2002; Wu et al., 2020). Additionally, it can directly break down pollutants, achieving soil remediation (Al-Shara et al., 2020; Guo et al., 2014). However, prolonged electrokinetic treatment leads to a gradual weakening of the electric current, which reduces remediation efficiency. To counteract this, Barba et al. (2017) maintained the electrokinetic effect by periodically reversing the electrode polarity. Similarly, Alshawabkeh et al. (2004) applied methods such as ion supplementation, voltage increase, and polarity reversal to mitigate current attenuation. Despite these efforts, the decline in electrokinetic effects over time remains a challenge.

Phytoremediation, a sustainable and eco-friendly technology, is also employed for the treatment of petroleum-contaminated soils. Vegetation can help overcome some of the limitations of electrokinetic remediation. Plants degrade soil contaminants through mechanisms such as phytoextraction, phytostabilization, phytodegradation, and root exudation (Babu et al., 2021). Plants with extensive fibrous root systems occupy a large soil volume, regulate soil moisture, and prevent soil erosion on a macroscopic scale (Ndimele, 2010). Moreover, roots create pores that improve soil connectivity and diffusivity, enhancing transport processes and soil structure (Yildiz et al., 2018). Plant roots also exude organic acids such as formic, acetic, and malic acids, which lower soil pH (Marschner et al., 1987). However, phytoremediation alone has a lengthy remediation cycle and is effective primarily within the root zone (Yu et al., 2021).

Combining phytoremediation with electrokinetic remediation offers a solution to the limitations of phytoremediation, such as its long duration and limited reach, while also mitigating the soil damage caused by the electrokinetic process (Mulati et al., 2023). Existing research has demonstrated the synergistic effects between electrokinetic remediation and plants in various contexts. Huang et al. (2019) found that electrokinetic-phytoremediation can overcome contaminant depletion zones created by slow diffusive transport. Sánchez et al. (2020a) reported that electrokinetic-assisted phytoremediation reduced water evaporation and enhanced the degradation of the herbicide atrazine. Wu et al. (2020) showed that coupling electrokinetics with plants could slow current attenuation and increase n-hexadecane degradation rates. Yuan et al. (2024) demonstrated that electrokinetic remediation with ryegrass effectively removed mixed organic and heavy metal pollutants from soil. However, most studies have focused on identifying the manifestations of electrokinetic-plant synergy and elucidating the underlying mechanisms, with limited research on the influence of plant density on synergistic effects related to ion dynamics, moisture characteristics, and current maintenance.

This study aims to address this gap by investigating how tall fescue planting density influences the synergistic effects in electrokinetic-phytoremediation for petroleum-contaminated soil. The impact of various planting densities on parameters such as electric current, soil pH, ion concentrations, moisture characteristics, and soil colloidal structure was assessed during the remediation process. The findings provide a theoretical framework for further optimizing this combined technology to improve the remediation efficiency of contaminated soils.

Compared with prior studies that mainly demonstrated qualitative benefits of electrokinetic (EK)–plant coupling, this work treats planting density as an explicit and adjustable design variable and connects density-dependent remediation outcomes to (i) sustained electrical current and ion dynamics and (ii) colloidal-chemical indicators (zeta potential and electric double layer thickness) that reflect soil structural stability under an electric field. This integrated perspective provides a mechanistic basis for optimizing EK-assisted phytoremediation in petroleum-contaminated soils.

We hypothesized that planting density regulates EK–phytoremediation through a trade-off: increasing density enhances root–soil contact and rhizosphere stimulation, which helps maintain ionic conductivity and colloidal stability and thus sustains current, whereas excessive density increases intraspecific competition and nutrient depletion, weakening these benefits. Accordingly, our objectives were to (1) quantify density-dependent petroleum removal and synergistic enhancement, and (2) elucidate how planting density modulates current, pH, ion distributions, moisture retention, and colloidal properties during EK-assisted phytoremediation.

## 2 Materials and methods

### 2.1 Soil and plant species

Soil was collected from farmland near Shenyang, China, at a depth of 0–20 cm. After air-drying, small gravel, plant debris, and other non-soil materials were removed, and the soil was sieved through a 2 mm mesh. The processed soil was stored at low temperature and aerated before use. The main physicochemical properties of the soil are provided in **Supplementary Table 1**. The petroleum used in the experiment was crude oil from the Liaohe Oilfield in Panjin, with a density of 0.903 g/cm^3^ at 25 °C and a pour point of 26.0 °C. Petroleum-contaminated soil was artificially prepared by dissolving crude oil in chloroform and thoroughly mixing it with clean soil. The mixture was left in a ventilated area for two weeks, turning daily to ensure complete evaporation of chloroform. Afterward, the contaminated soil was combined with unpolluted soil and sieved for homogeneity, resulting in a final petroleum concentration of 3% (w/w).

Tall fescue (*Festuca arundinacea*) was used in this experiment, as it has a well-developed root system, strong vitality, and grows quickly. In addition, it can grow to its adult size with its oil-degrading function within a short period (Liu et al., 2013). Tall fescue seeds were sourced from Shandong Ruihe Seed Industry Co., Ltd. (China).

### 2.2 Experimental setup and treatment groups

**Supplementary Table 2** outlines the experimental treatments, which include an electrokinetic-only control (EK), four electrokinetic + plant treatments with varying planting densities (EK-P1 to EK-P4, with densities of 0.5, 1, 2 and 3 plants/cm^2^, corresponding to 5,000, 10,000, 20,000, and 30,000 plants/m^2^, respectively), a plant-only treatment (P), and an untreated control (CK). All treatments were established in three independent replicate units (n = 3), where each replicate consisted of one soil container operated as an independent remediation unit. For each treatment, 4 kg of the prepared petroleum-contaminated soil was placed into a rectangular container and thoroughly mixed with water to achieve a soil moisture content of 21% (approximately 70% of field capacity). Graphite electrodes were inserted into the soil at fixed positions within the container. Tall fescue seeds (with empty husks removed) were surface-sterilized in a 20% (v/v) NaClO solution for 10 minutes, then rinsed five times with sterile water. The seeds were soaked in water for 24 hours and placed in Petri dishes in an incubator for germination. When the shoots grew to 5 mm, the seeds were transplanted into clean soil and cultivated for 20 days. Subsequently, plants with consistent growth status were selected and transplanted into contaminated soil in accordance with the experimental planting density. The electrodes in each container were connected to a DC power supply, with periodic polarity reversal every 30 minutes. The experimental setups were placed in a greenhouse with artificial lighting and temperature control, maintaining a light/dark cycle of 16/8 hours. Daytime temperature was approximately 25 °C, and nighttime temperature was about 18 °C. Deionized water was added by weight every three days to maintain consistent soil moisture content.

**Figure 1:**
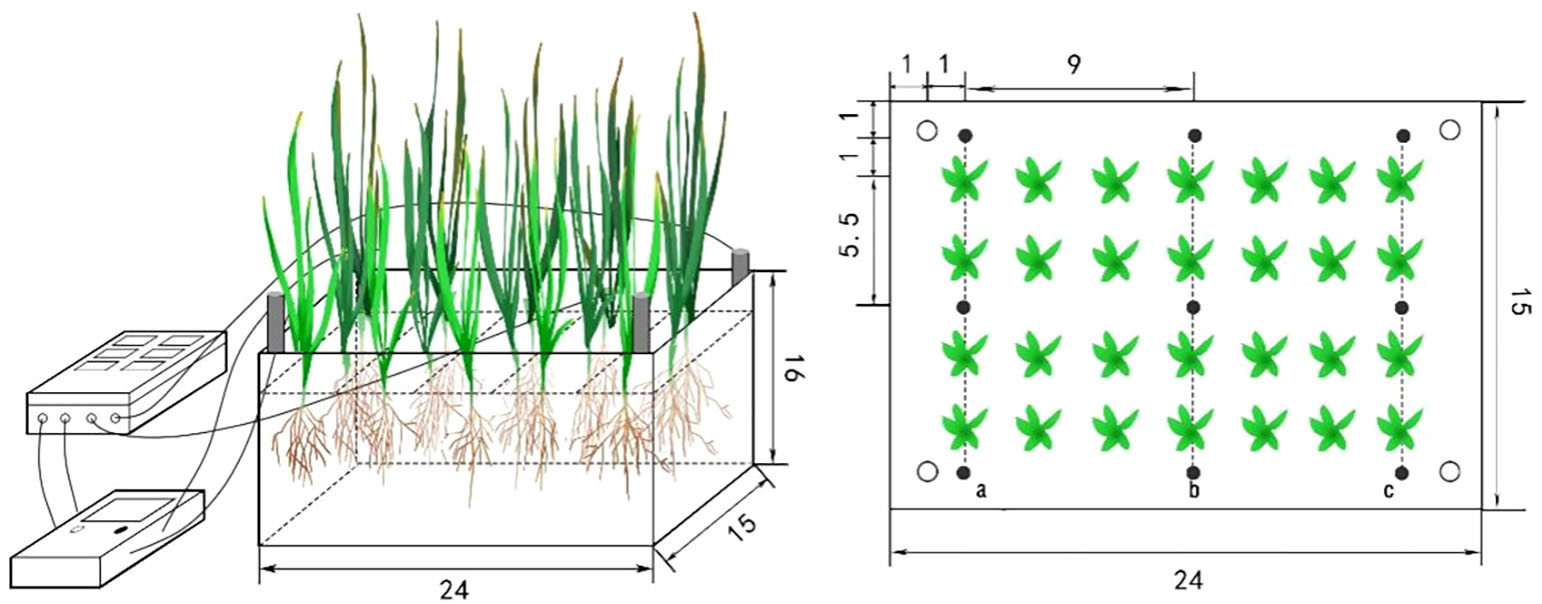
Experimental setup of the electrokinetic-assisted phytoremediation system (dimensions in cm).

As illustrated in **Figure 1**, the experimental setup included a DC power supply, a custom-built current monitoring device, and a soil sample container. Each container measured 22 cm × 15 cm × 16 cm and was equipped with four graphite electrodes (15 cm length × 0.8 cm diameter) to generate a uniform electric field of 1 V/cm across the soil, multiple studies on petroleum-contaminated soils confirm that a 1 V/cm field with periodic polarity reversal over extended durations (e.g. 60 days) improves total petroleum hydrocarbon (TPH) degradation (Abou-Shady and El-Araby, 2024). The polarity of the electrodes was reversed every 30 minutes. All containers were connected in parallel to ensure consistent electric field strength across each setup. The soil in each container was divided into three sections (positions a, b, and c), as depicted in the figure, following the method of Wu et al. (2020). The experiment was conducted over a 60-day period. At the conclusion of the experiment, At the conclusion of the experiment, soil and plant samples were collected from each replicate unit. For analyses of soil water retention, zeta potential, electric double layer thickness, and petroleum degradation rate, samples from positions a, b, and c were combined. For water-soluble ion and pH analysis, samples from positions a and b (electrode zones) were pooled, while the sample from position c (middle zone) was kept separate. Since no electric field was applied in the P treatment, there were no electrode or non-electrode regions. Therefore, samples from points a, b, and c were thoroughly mixed to represent the overall parameter measurements for this treatment.

### 2.3 Analytical methods

#### 2.3.1 Electric current

The electric current in each electrokinetic system was recorded using a custom current logging device. Measurements were taken every three days. After rewatering the soil, the system was allowed to stabilize for 30 minutes before recording the current value.

#### 2.3.2 Soil pH

Soil pH was measured by shaking 4 g of air-dried soil with 10 mL of deionized water (soil-to-water ratio 1:2.5) for 30 minutes. The pH of the resulting suspension was then measured using a pH meter (PHS-3E).

#### 2.3.3 Water-soluble ions

The concentrations of water-soluble cations (Ca^2+^, Mg^2+^, Na^+^, K^+^) in the soil were determined by ion chromatography. For each sample, 4 g of sieved soil (< 0.85 mm, 20-mesh) was placed in a centrifuge tube with 20 mL of deionized water (soil-to-water ratio 1:5). The mixture was shaken at 150 rpm for 30 minutes and then centrifuged at 4000 rpm for 5 minutes. The supernatant was filtered and analyzed using an ion chromatograph (Thermo ICS-600).

#### 2.3.4 Cationic exchange capacity of plant roots

Dried root powder (0.1000 g) of tall fescue was placed in 200 mL of 1 mol/L KCl solution (pH = 7.0). The mixture was stirred for 5 minutes until the roots settled. The roots, containing the exchanged potassium, were placed in a funnel and rinsed with deionized water until no chloride ions were detected (using AgNO_3_). The cleaned roots were then placed in 200 mL of 0.1 mol/L HCl and stirred for 5 minutes. The concentration of K^+^ in the solution was measured using atomic absorption spectrometry (AAS, Varian, AA240). The CEC of the root system was calculated as described by Torrent and Barrón (2008).

#### 2.3.5 Soil water retention curve

Soil water retention (moisture characteristic) curves were determined using a pressure membrane apparatus. After remediation, 40 g of naturally air-dried soil from each treatment was packed into a stainless-steel ring (soil core cutter). The soil in the ring was saturated by placing the ring in deionized water until the soil was fully saturated (with the water level kept below the top of the soil). The ring with saturated soil was then placed on a ceramic plate in the pressure membrane apparatus. The chamber was sealed, and pressure was increased to a specified value. After equilibrium was reached (no further outflow of water, approximately 24 hours), the soil sample was removed, and its moisture content was determined gravimetrically. Each sample was measured in triplicate. The resulting data points were fitted using MATLAB to obtain the soil water retention curve for each treatment.

#### 2.3.6 Zeta potential

Soil colloid zeta potential was measured using a zeta potential analyzer (Brookhaven Zeta). A 1 g portion of soil (sieved to < 150 µm, 100-mesh) was suspended in 25 mL of deionized water. The suspension was shaken for 30 minutes, followed by ultrasonication for 30 minutes to disperse the particles. The sample was allowed to settle briefly, and an aliquot of the upper suspension (0–2 cm below the surface, containing the finest particles) was taken for zeta potential measurement. The average of five measurements was reported as the zeta potential for each sample.

#### 2.3.7 Soil colloid double layer thickness

The thickness of the soil colloidal electric double layer (Debye length) was determined using a magnesium exchange method. Soil samples were treated repeatedly with 0.5 mol/L Mg (NO_3_)_2_ solution with shaking to saturate the cation exchange sites with Mg^2+^. The samples were then rinsed with deionized water (with shaking) until the Mg^2+^ concentration in the rinse water was below 0.5×10–^4^ mol/L. The samples were dried, ground, and passed through a 60-mesh sieve. Then, 2.5 g of the Mg-saturated soil was added to 100 mL of 1.1×10–^4^ mol/L Mg (NO_3_)_2_ solution and shaken for 24 hours to reach ion exchange equilibrium. The Mg^2+^ concentration in the suspension was measured using an atomic absorption spectrophotometer. The electric double layer thickness (κ^–1^) was calculated using the Debye length formula:


$$\frac{1}{\kappa }=\sqrt{\frac{\epsilon RT}{8\pi F^{2}Z_{i}C_{i}}}$$

where *C_i_* is the concentration of ionic species *i* in the 2:1 (electrolyte (Mg(NO_3_)_2_ in this case), *Z_i_* is the valence of ion *i* (for Mg^2+^, Z = 2), *F* is the Faraday constant (96,485 C/mol), *ϵ* is the permittivity of water (6.96×10–^10^ C^2^/J·dm), *R* is the gas constant (8.314 J/mol·K), and *T* is the absolute temperature (298 K).

#### 2.3.8 Petroleum degradation rate

The total petroleum hydrocarbon (TPH) removal was determined by Soxhlet extraction with dichloromethane followed by gravimetric analysis. This gravimetric method quantifies bulk extractable hydrocarbons and does not resolve compositional changes (e.g., aliphatic versus aromatic fractions). The TPH degradation rate (removal efficiency) was calculated as the percentage of TPH mass removed from the soil after 60 days, relative to the initial TPH mass.

### 2.4 Statistical analysis

All results are presented as mean ± standard deviation (SD) from three independent replicate units (n = 3), unless otherwise stated. Prior to hypothesis testing, normality and homogeneity of variance were checked using the Shapiro–Wilk test and Levene’s test, respectively. For endpoint variables (e.g., 60-day TPH removal, zeta potential, electric double layer thickness, soil water-retention parameters, plant biomass, and root CEC), differences among treatments were evaluated using one-way ANOVA followed by Tukey’s HSD *post hoc* test (*p* < 0.05). When ANOVA assumptions were violated, the Kruskal–Wallis test with Dunn’s multiple-comparison correction was applied. For time-course data (electric current and water-soluble ion concentrations), linear mixed-effects models were used with treatment, time, and (when applicable) zone as fixed effects and replicate as a random effect; *post hoc* comparisons were adjusted using the Tukey method. Correlations between TPH removal and key predictors (ion concentrations, zeta potential, plant height, and root length) were assessed using Pearson or Spearman correlation depending on data distribution (*p* < 0.05).

## 3 Results and discussion

### 3.1 Effect of planting density on remediation performance

**Figure 2:**
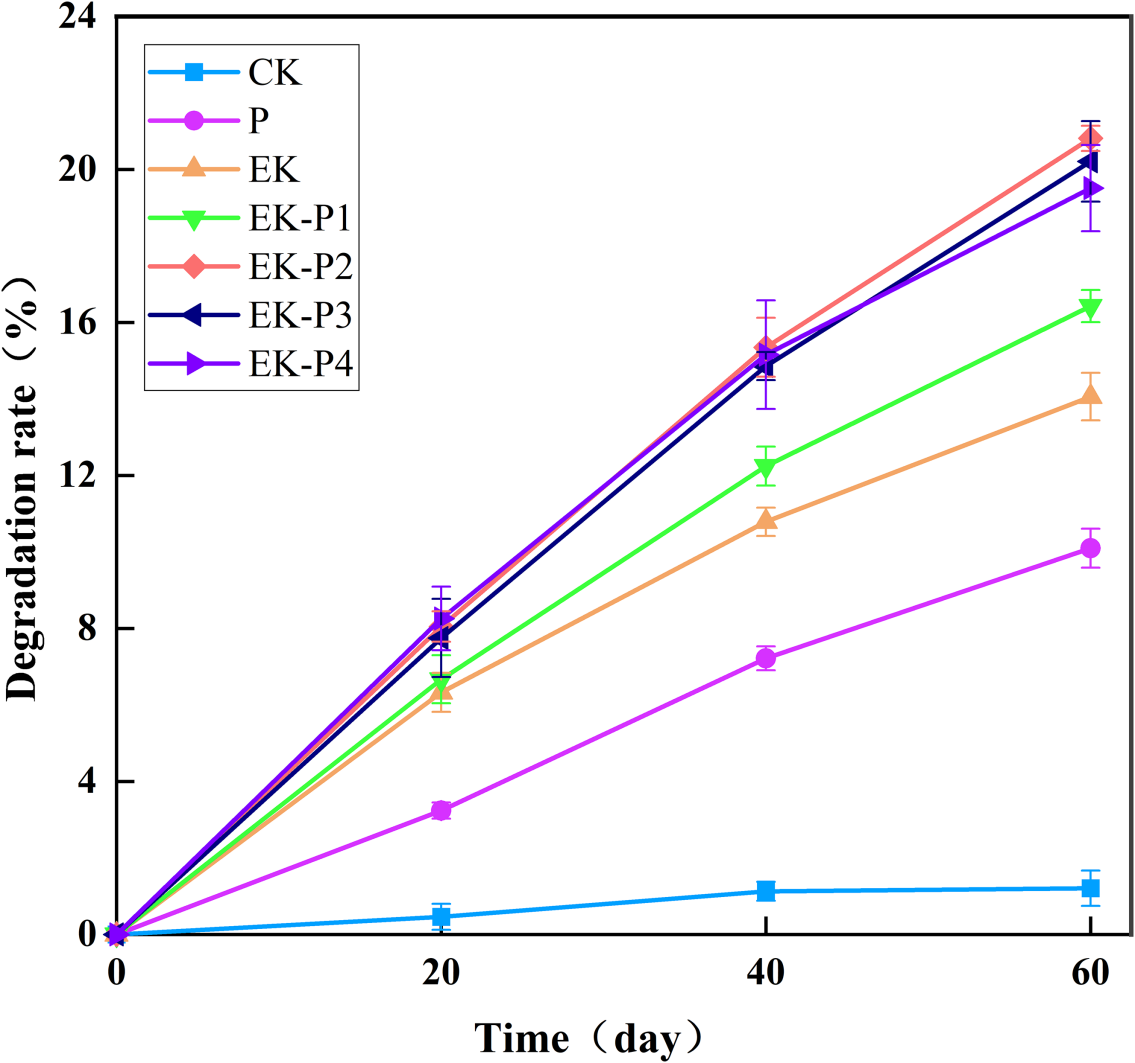
TPH removal (%) after 60 days of remediation for each treatment (mean ± SD, n = 3). Different letters indicate significant differences among treatments (one-way ANOVA with Tukey’s HSD, p < 0.05).

**Figure 2** presents the TPH degradation rates after 60 days for each treatment. The degradation rate of EK-P (1-4) increases with time, but the increase rate decreases. The EK-P2 treatment achieved the highest average TPH removal at 20.81%. The EK-P3 and EK-P4 treatments followed with slightly lower removal rates of 20.21% and 19.51%, respectively. EK-P1 achieved a removal rate of 16.43%, while the EK-only (EK) treatment reached 14.06%. The P (plant-only) treatment showed a TPH removal rate of 10.1%, and natural attenuation in the control (CK) resulted in only 1.21%. In the EK-P (1-4) treatment, the degradation rate was notably higher than that in the EK treatment and the P treatment which indicates a synergistic effect between electricity and plants. To quantify the synergistic enhancement of EK–phytoremediation, we used a bounded highest-single-process (HSP) metric: SE = RE(EK-P) − max [RE(EK), RE(P)], where RE denotes removal efficiency. Because RE(EK) exceeded RE(P) in our system, SE is equivalent to RE(EK-P) − RE(EK). Accordingly, the synergistic enhancement was 2.37, 6.75, 6.15, and 5.45 percentage points for EK-P1 through EK-P4, respectively, indicating a non-linear density response with the maximum enhancement at 1 plant/cm^2^. Consistent with this, EK-P2 was not significantly different from EK-P3 but both were significantly higher than EK and P (p < 0.05). Because TPH was quantified as bulk extractable hydrocarbons, the observed reduction reflects the combined effects of biodegradation, volatilization, and electrochemical transformation; compositional changes (aliphatic versus aromatic fractions) were not resolved in the present study. Moreover, the correlation between TPH removal efficiency and cation concentration, zeta potential, root length, plant heigh reached a significant level (*p* < 0.05) (**Supplementary Table 3**). This means that plant height, root length, zeta potential, and cation concentration are significant influencing factors for TPH removal efficiency. These findings highlight that integrating tall fescue with electrokinetic remediation significantly enhances petroleum degradation, due to the synergistic interactions between the plants and the electric field.

### 3.2 Effect of planting density on sustaining electric current

#### 3.2.1 Electric current and pH

**Figure 3:**
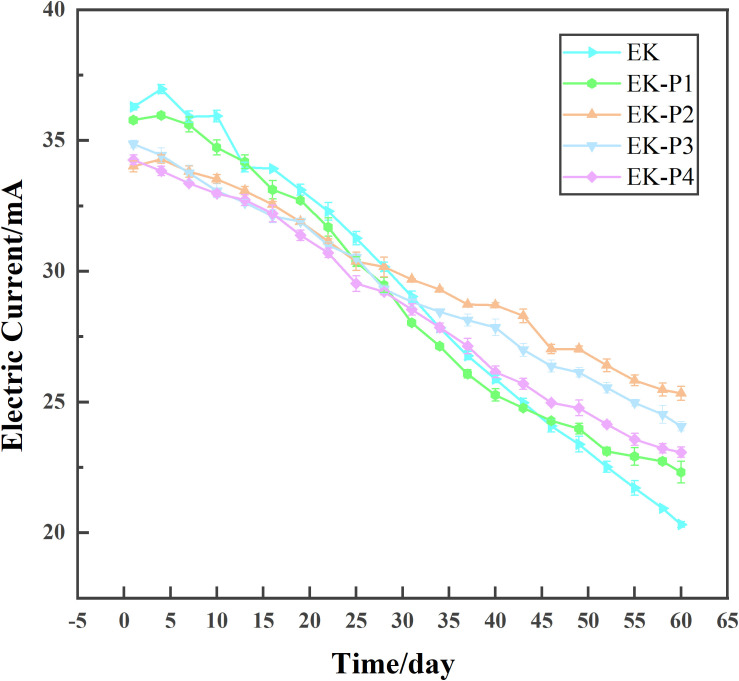
Electric current variation over time in the EK and EK-P1–EK-P4 treatments (mean ± SD, n = 3).

Across all experimental treatments, the electric current exhibited a decline over time. As illustrated in **Figure 3**, the EK (electrokinetic-only) group demonstrated the fastest current decay, followed by EK-P1, EK-P4, and EK-P3, with EK-P2 showing the slowest decline. In the EK group, the current decreased gradually during the first ~20 days, then dropped sharply between 20–40 days, followed by a slowdown from 40–60 days, resulting in an overall reduction of 44.01% by day 60. The EK-P1 group followed a similar trend to EK but with a final reduction of 37.63%. In contrast, the EK-P2, EK-P3, and EK-P4 groups exhibited slower current decay than EK, with reductions of 28.62%, 30.98%, and 32.63%, respectively, after 60 days. These results suggest that plant presence enhanced the retention of electrokinetic current (Mary M, 2002). At the lowest planting density (EK-P1), the effect on current retention was modest. Increasing planting density led to enhanced current retention (EK-P2 exhibited the best maintenance), though further increases in density (EK-P3 and EK-P4) resulted in greater current decay, indicating a threshold effect of planting density on current maintenance. Factors influencing current intensity include soil water-soluble ion content, soil structure, and moisture characteristics. Consequently, varying planting densities of tall fescue affected these parameters, resulting in the observed differences in current (Kumar et al., 1995). Mixed-effects modeling indicated that both planting density and its interaction with time significantly influenced current decay (*p* < 0.05).

The higher TPH removal observed in the planted electrokinetic treatments can be attributed to concurrent improvements in key electrokinetic controlling factors. Compared with EK alone, the presence of tall fescue moderated soil pH, slowed the depletion of water-soluble ions, and preserved more favorable soil moisture characteristics and colloidal stability, all of which contributed to sustaining electrical current and enhancing petroleum attenuation. Although the electric field with periodic polarity reversal can stimulate water and ion redistribution toward roots, we did not directly quantify nutrient fluxes or root exudate profiles; therefore, mechanistic interpretations that involve rhizosphere acidification and reduced Ca^2+^/Mg^2+^ precipitation are supported primarily by our measured pH/ion patterns and by prior literature, rather than direct measurements in this study. Furthermore, the marginal improvement in remediation performance at higher planting densities likely reflects a trade-off between increased root density (beneficial for EK–plant coupling) and intraspecific competition and nutrient uptake at excessive densities (which may reduce ion availability and plant vigor). We therefore use ‘synergistic enhancement’ to refer to the quantitative SE metric defined above, and we interpret mechanistic links conservatively as plausible drivers rather than directly proven causal pathways.

As presented in **Table 1**, soil pH decreased after 60 days of remediation across all electrokinetic (EK and EK-P) treatments. The EK treatment (without plants) exhibited the smallest decrease in pH, while treatments EK-P1 through EK-P4 showed progressively larger reductions as planting density increased. Electrochemical reactions at the electrodes play a critical role in the electrokinetic effect and significantly influence soil pH changes (Wang et al., 2016). In this study, inert graphite electrodes were employed, with polarity reversed every 30 minutes. Under these conditions, the primary anodic reaction is 4 OH^–^ – 4 e^–^ → O_2_↑ + 2 H_2_O, and the primary cathodic reaction is 2 H^+^ + 2 e^–^ → H_2_↑ (Alshawabkeh et al., 2004; O'Connor et al., 2003). Notably, H^+^ ions move approximately 1.7 times faster than OH^–^ in water. Periodic reversal of electrode polarity mitigated the accumulation of H^+^ and OH^–^ ions in the anodic and cathodic zones over time, resulting in only a modest net decrease in soil pH across the EK system (Saini et al., 2021). In the planted treatments (EK-P1–EK-P4), root activity further influenced pH. Root exudates, including small organic acids, enhance soil buffering capacity and locally acidify the rhizosphere. As a result, soil pH in planted treatments was lower than in the EK treatment, with pH steadily decreasing as planting density increased, approaching neutral conditions. The highest planting density (EK-P4) produced the lowest pH, although it did not differ significantly from EK-P2 (Sánchez et al., 2020a). Soil pH directly impacts ion solubility, the zeta potential of soil colloids, and colloidal structural stability, thereby indirectly affecting electrical current during the remediation process.

**Table 1:** Soil pH of each treatment after 60 days of remediation.

Position	EK	EP1	EP2	EP3	EP4	P1	CK
a, b	7.57 ± 0.12	7.47 ± 0.23	7.31 ± 0.11	7.19 ± 0.15	7.15 ± 0.21	7.35 ± 0.11	7.69 ± 0.22
c	7.62 ± 0.12	7.43 ± 0.16	7.28 ± 0.14	7.22 ± 0.15	7.18 ± 0.16		

Values are mean ± SD (n = 3). Two positions were measured for EK treatments: the electrode zones (a,b) and the middle zone (c)For treatments P and CK without electrodes, a single bulk sample was measured.

#### 3.2.2 Distribution of water-soluble cations in soil

Ion migration under the electric field generates the electrokinetic current. In the polarity-reversing electric field, cations move back and forth between the anode and cathode. Depending on their interactions with electrolysis products or other soil ions, some cations may precipitate or become immobilized, resulting in changes in their soluble concentrations over time.

**Figure 4:**
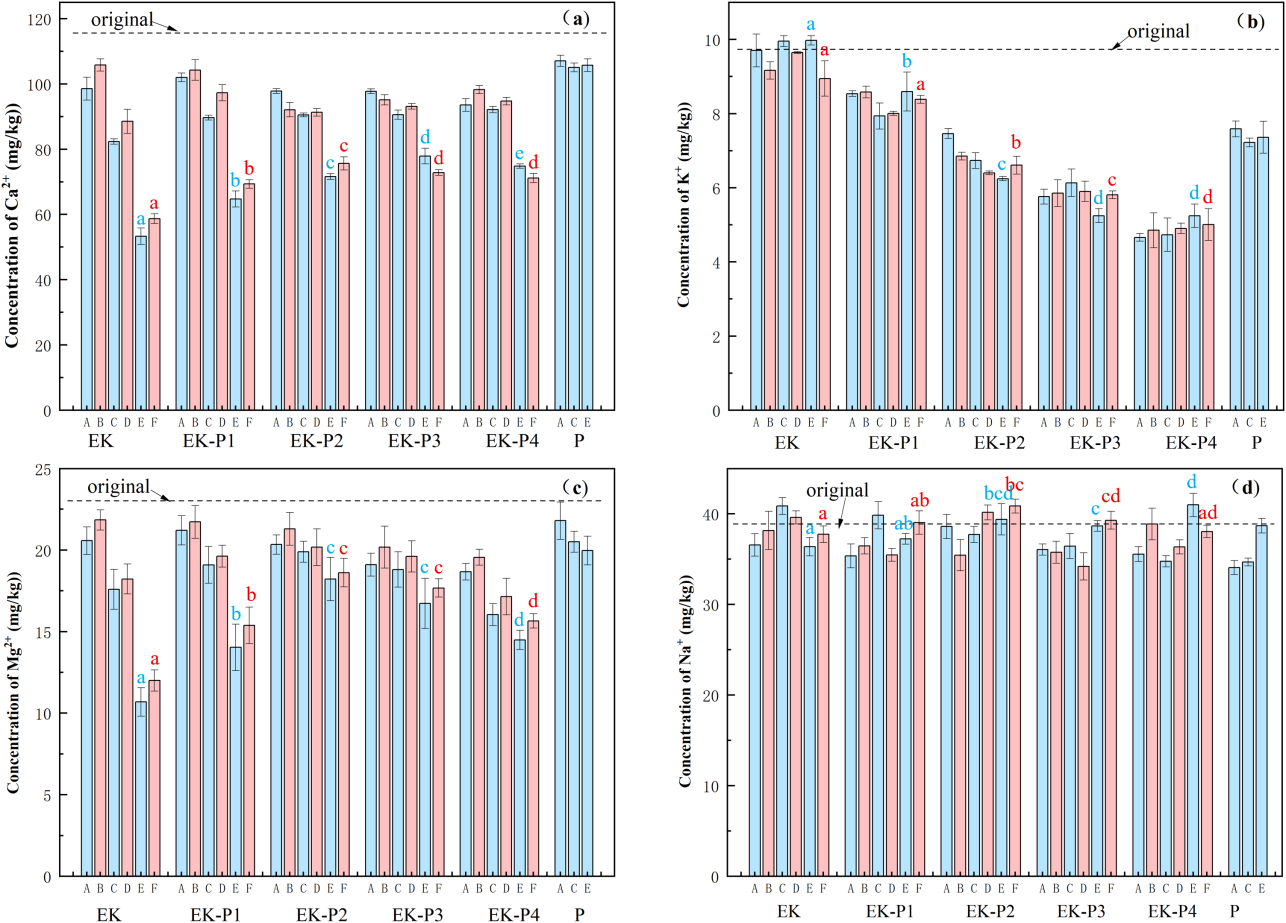
Variation in water-soluble ion concentrations [**(a)** Ca^2+^; **(b)** K^+^; **(c)** Mg^2+^; **(d)** Na^+^] over time in each treatment. The dashed line represents the initial ion concentration in the soil. (A, C, E) show samples from the electrode zones (combined positions a+b) at 20, 40, and 60 days, respectively, while (B, D, F) represent samples from the middle zone (position c) at 20, 40, and 60 days. Lower-case letters indicate significant differences between groups at the end of the experiment (*p* < 0.05).

**Figures 4a, c** display the concentrations of water-soluble Ca^2+^ and Mg^2+^, respectively, over the 60-day period. In all EK and EK-P1–EK-P4 treatments, the concentrations of Ca^2+^ and Mg^2+^ in both the electrode regions (combined a+b) and the middle region (c) decreased over time. In the P (plant-only) group, concentrations of Ca^2+^ and Mg^2+^ initially decreased by day 20 but remained relatively stable thereafter. In the EK group, Ca^2+^ and Mg^2+^ concentrations declined slowly during the first 20 days, followed by a more rapid decrease from days 40 to 60. This pattern is likely due to the shuttle of Ca^2+^ and Mg^2+^ between the electrodes under the applied electric field. In an alkaline environment, these ions readily form precipitates, leading to a decrease in their soluble concentrations as the remediation progresses (Cheng et al., 2017). In the planted EK-P treatments, the decline in Ca^2+^ and Mg^2+^ was less pronounced than in EK, with concentrations of water-soluble Ca^2+^ and Mg^2+^ remaining higher at 60 days compared to the EK-only treatment. The EK-P1 treatment (lowest density) exhibited a trend similar to EK, showing the greatest loss of Ca^2+^ and Mg^2+^, while EK-P2 showed the least loss, with the highest residual concentrations of these ions. Increasing planting density further to EK-P3 and EK-P4 did not result in continued reduction of ion loss; in fact, final concentrations of Ca^2+^ and Mg^2+^ in EK-P4 were slightly lower than those in EK-P2. These results demonstrate that the planted EK-P systems (especially EK-P2) retained higher soluble Ca^2+^ and Mg^2+^ than the EK-only treatment after 60 days. Mechanistically, two plausible (inferred) processes may contribute. First, root activity, potentially including exudation of low-molecular-weight organic acids, can acidify the rhizosphere, which would weaken cathodic alkalinization and thereby reduce Ca^2+^ and Mg^2+^ precipitation (Wang and Kuzyakov, 2024). Second, transpiration-driven mass flow may draw water and dissolved ions toward the root zone, potentially retaining Ca^2+^ and Mg^2+^ near roots and limiting their interaction with electrochemical reaction products. However, root exudate chemistry and solid-phase Ca/Mg precipitates were not directly quantified in this study; therefore, these pathways are presented as hypotheses supported by our measured pH/ion trends and prior literature, rather than as demonstrated causal mechanisms. At the highest planting densities, water-soluble Ca^2+^ and Mg^2+^ concentrations were slightly lower than at moderate densities, likely because Ca and Mg are essential plant nutrients that vegetation absorbs in small amounts (Wang and Kuzyakov, 2024).

**Figure 4d** shows the water-soluble Na^+^ concentration. In the EK group, Na^+^ concentrations in both the electrode and middle regions did not change significantly, with only a slight decrease by day 60. Na^+^ remains soluble and does not form precipitates; variations in Na^+^ are primarily due to redistribution among soil phases (Lieser, 1969). In the EK-P1–EK-P4 groups, Na^+^ initially decreased slightly, then increased, ultimately returning to approximately the initial concentration, indicating that planting density had no significant net effect on Na^+^ levels. The P group followed a similar pattern. This behavior can be explained by plants initially absorbing some Na^+^ during early growth as an osmoticum to help balance osmotic pressure (Sánchez et al., 2020b). By day 60, Na^+^ concentrations tended to return toward the initial level. This rebound may reflect reduced net Na^+^ uptake later in the growth period and redistribution of Na^+^ between plant tissues and the soil solution (Almeida et al., 2017). However, plant ion fluxes and root senescence were not directly measured, so this explanation is discussed as plausible rather than demonstrated. Ultimately, no significant differences in water-soluble Na^+^ concentrations were observed among the treatments.

After 60 days of remediation, the total concentration of water-soluble cations (Ca^2+^ + Mg^2+^ + K^+^ + Na^+^) in the EK-P1, EK-P2, EK-P3, and EK-P4 treatments was higher than in the EK treatment by approximately 14.77, 24.37, 18.23, and 12.52 mg/kg, respectively. The low planting density (EK-P1) was insufficient to significantly counteract the ion loss induced by electrokinetics. Very high planting densities (EK-P3, EK-P4) did not further mitigate ion loss; instead, greater plant uptake of nutrients such as K^+^ and Mg^2+^ at high densities reduced the soil’s water-soluble ion content. Among the tested densities, EK-P2 (1 plant/cm^2^) was most effective in retaining soluble ions, exhibiting the slowest decline in ion concentrations, which remained the highest in this treatment. Soil water-soluble ion concentration is a critical factor influencing electrokinetic current, as higher ion concentrations result in stronger currents. Therefore, maintaining higher soluble ion levels contributed to better sustenance of the electrokinetic current at the optimal planting density.

#### 3.2.3 Soil structure (Zeta potential and double layer thickness)

**Figure 5:**
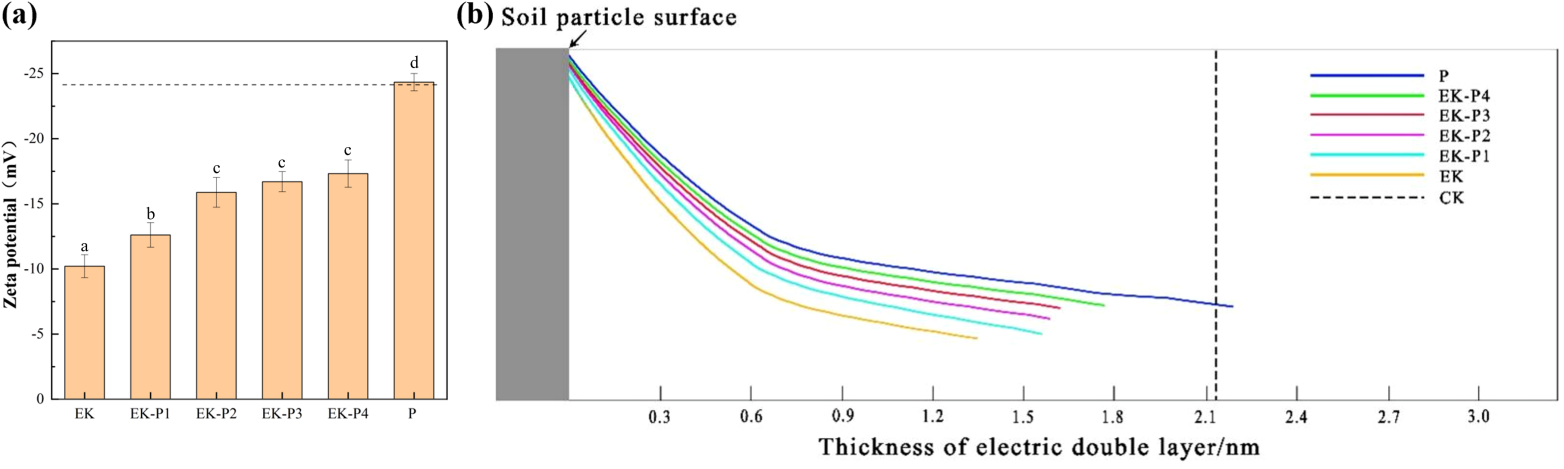
**(a)** Zeta potential of soil colloids after 60 days of remediation for the EK, EK-P1–EK-P4, and P treatments. **(b)** Electric double layer thickness of soil colloids after 60 days of remediation for the EK, EK-P1–EK-P4, and P treatments (mean ± SD, n = 3). Different letters indicate significant differences (*p* < 0.05).

Changes in soil pH and soluble ion concentrations can impact the stability of the soil colloidal structure, which in turn affects the electric current. Zeta potential represents the electric potential at the shear plane of soil colloids, and together with the thickness of the electric double layer (EDL), it indicates the dispersion or flocculation state of soil colloids (Rozas and Castellote, 2015). **Figures 5a, b** present the zeta potential and bilayer thickness of the soil, respectively.

After 60 days of remediation, the EK treatment exhibited substantial changes in colloidal properties: the zeta potential became less negative, increasing from an initial -24.34 mV to -9.87 mV (a 63.9% increase toward zero), and the EDL thickness (Debye length) decreased from 2.11 nm to 1.35 nm. This likely occurred due to continuous electrokinetic forces causing ions to precipitate and adsorb onto the surfaces of soil colloids, thereby shielding the negative surface charges. As a result, the zeta potential increased (i.e., the colloids became less negatively charged) and the double layer collapsed, transitioning the colloids from a dispersed to a flocculated/aggregated state, ultimately damaging soil structure (Cheng et al., 2017).

In the EK-P1–EK-P4 treatments with plants, changes in zeta potential and EDL thickness over 60 days were less pronounced. In the EK-P1 group, the zeta potential increased from -27.34 mV to -15.98 mV (a 41.55% rise) and the EDL thickness decreased from 2.11 nm to 1.55 nm. In EK-P2, the zeta potential increased from -27.34 mV to -17.98 mV (a 34.23% increase), and the EDL thickness reduced to 1.60 nm. In the EK-P3 and EK-P4 groups, the final zeta potentials were approximately -16.7 mV and -17.7 mV, with EDL thicknesses of 1.62 nm and 1.77 nm, respectively. In the P (plant-only) group, the zeta potential remained nearly unchanged, and the EDL thickness slightly increased, as no electric field was applied, leaving the soil structure unaffected. After 60 days, the zeta potentials in all EK-P groups were more negative (and the double layers thicker) compared to the EK group, suggesting that plant presence maintained the soil colloids in a more stable, dispersed state, thereby preserving soil structure more effectively (Wu et al., 2020). Root activity likely reduced ion precipitation on colloid surfaces, minimizing the shielding of negative charges, and alleviated the “ion focusing” effect of the electric field (Zhang et al., 2020). Consequently, colloidal properties in the planted treatments changed less compared to the EK-only treatment. These results demonstrate that plant presence mitigated electrokinetically induced colloid destabilization, as reflected by the more negative zeta potentials and thicker EDL compared with EK alone. Importantly, aggregate stability, pore-size distribution, and porosity were not directly measured; thus, statements about “soil structure protection” are inferred from these colloidal-chemical proxies rather than confirmed by direct physical-structure measurements. Related research can be one of the focuses of future research. This analysis underscores that plants play a critical role in protecting the soil colloidal structure. While increasing planting density further strengthened this protective effect, the benefits diminished at very high densities. The stability of the soil colloidal structure is essential for sustaining the electrokinetic current (Paillat et al., 2000).

#### 3.2.4 Soil moisture characteristics

The magnitude of the electrokinetic current is influenced not only by soil ions and colloidal structure but also by soil moisture conditions. The soil water retention curve, which describes the relationship between soil water content and matric potential, is a crucial parameter for understanding water and solute transport, as well as the availability of water and nutrients in soil.

**Figure 6:**
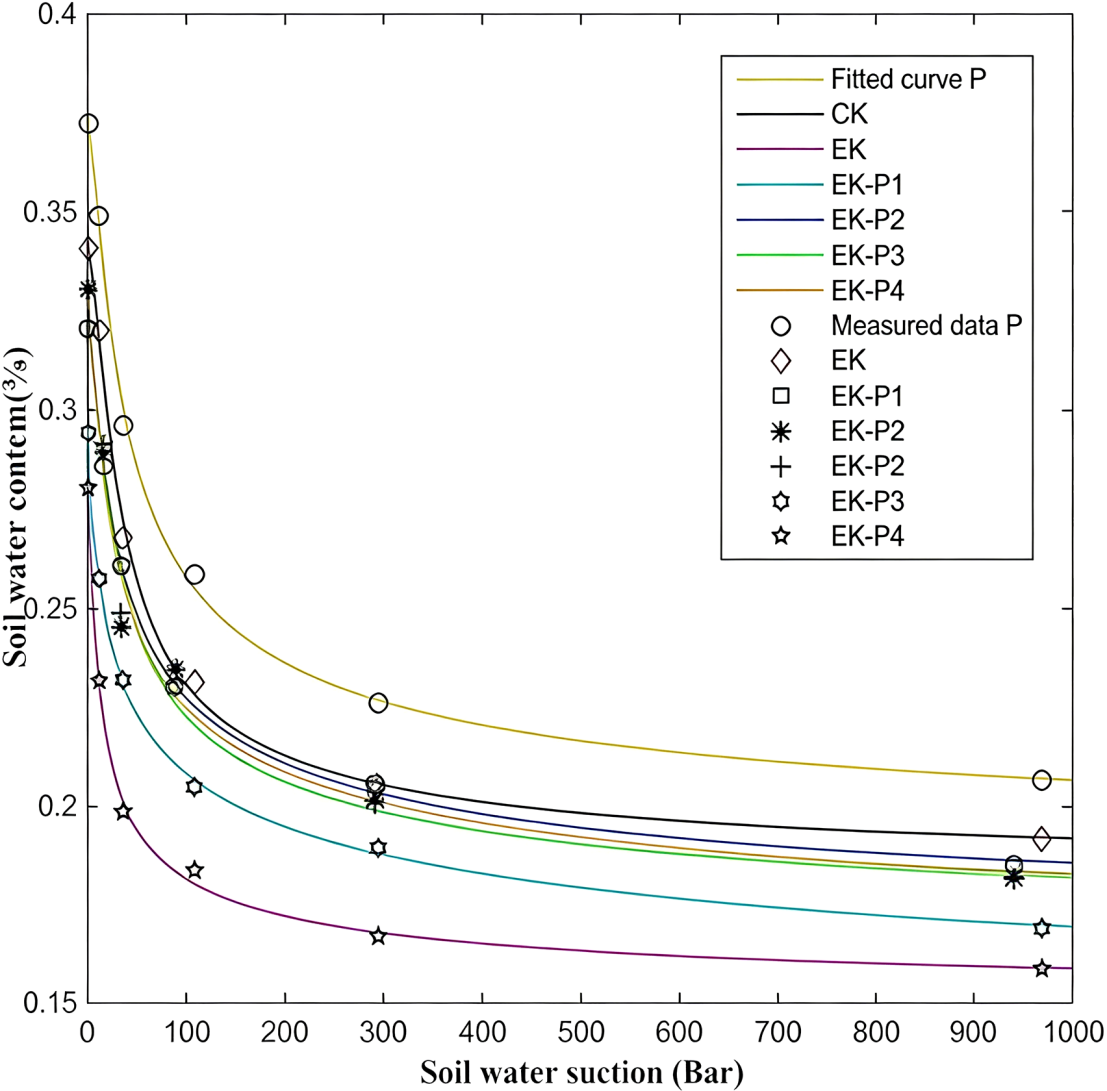
Soil water retention (moisture characteristic) curves of each treatment after 60 days of remediation (mean of three independent replicates; each curve point measured in triplicate).

As presented in **Figure 6**, after 60 days of electrokinetic remediation, the water retention curve for the EK treatment shifted downward relative to the untreated control (CK), indicating a reduction in soil water-holding capacity. This may reflect two primary processes. First, the application of a polarity-reversing electric field caused repeated cycles of drying and rewetting in the electrode regions. Combined with significant ion precipitation and loss, as well as disruption of soil aggregates, this led to decreases in both saturated water content and residual water content, thereby lowering the soil’s water-holding capacity (Nsabimana et al., 2020). Second, in the EK treatment, changes in soil zeta potential from approximately -24.5 mV to -9.9 mV, along with the compression of the electric double layer, caused colloidal aggregation. Precipitated ions clogged soil pores, reducing pore volume and increasing soil compaction (Chorom and Rengasamy, 1995). With the electric double layer compressed, the reduction in spacing between clay particles further diminished the soil’s ability to retain water (Nsabimana et al., 2020).

In contrast, the planted treatments showed improved soil moisture characteristics compared to the EK treatment. The downward shift of the water retention curve was more pronounced in EK-P1 (low density) and less so in EK-P3 and EK-P4, with EK-P2 showing the smallest shift, nearly maintaining the water retention characteristics of CK. The P (plant-only) treatment exhibited a slight upward shift in the water retention curve compared to CK, indicating improved water retention due to the presence of plant roots alone. These results demonstrate that vegetation mitigated the degradation of soil moisture properties caused by electrokinetics. Possible (inferred) contributors include root-induced changes in pore space, reduced salt/precipitate accumulation, and moderated zeta potential/EDL compression, which together may help preserve colloidal-chemical stability and water retention (Gui et al., 2021). However, we did not directly quantify aggregate stability, porosity, or root-induced pore formation; therefore, these explanations are discussed as plausible mechanisms rather than directly measured processes. Generally, plant presence promoted soil water retention, and increasing planting density beyond a certain threshold did not result in further significant improvements. Except for EK-P1, where the low density offered minimal protection to soil moisture characteristics, the EK-P2 to EK-P4 treatments showed comparable positive effects, with EK-P2 slightly outperforming EK-P3 and EK-P4. This suggests that the effect of planting density on soil water retention improvement increases initially and levels off once a certain density threshold is reached.

### 3.3 Synergy: effect of the polarity-reversing electric field on plant growth

**Figure 7:**
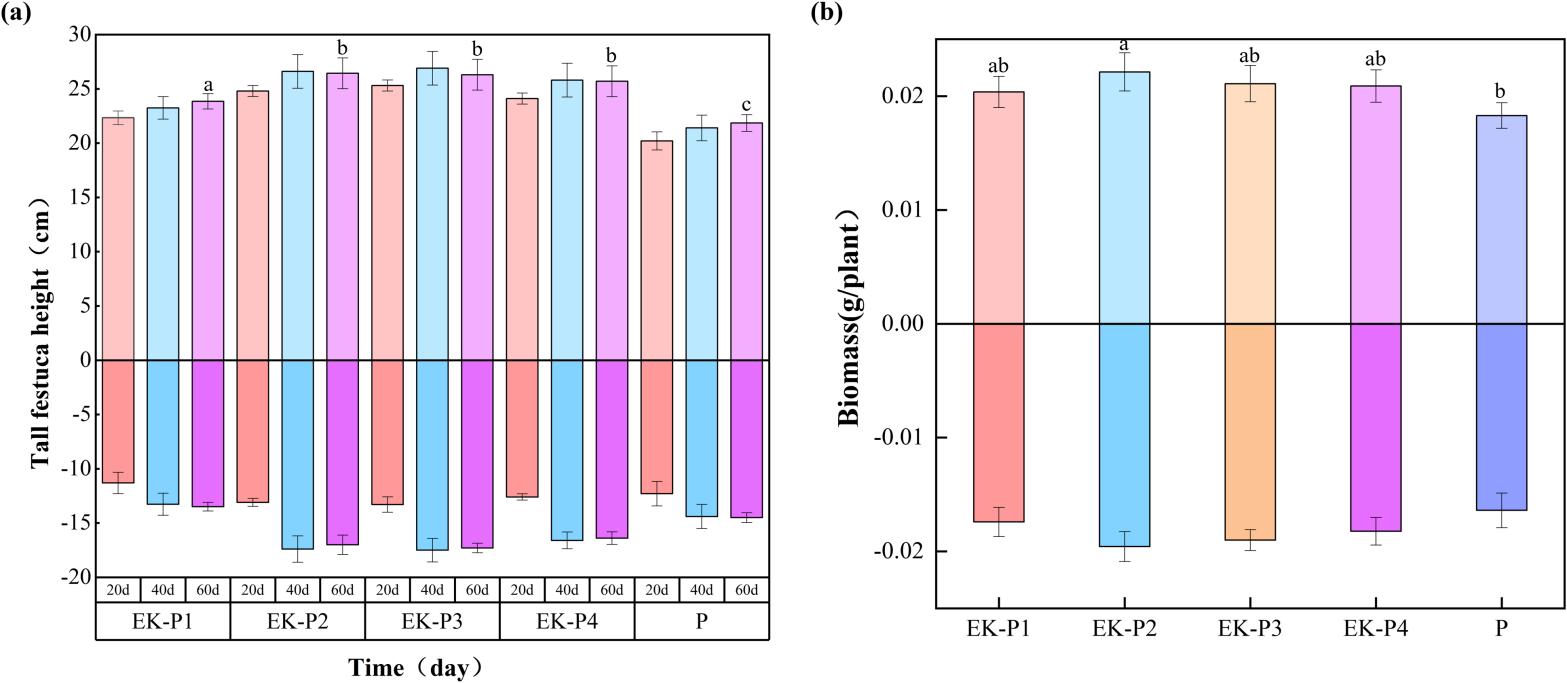
Growth of tall fescue in different treatments: **(a)** plant height over time; **(b)** plant biomass at 60 days (mean ± SD, n = 3). The part above coordinate axis represents shoot, and the part below coordinate axis represents the root. Different letters indicate significant differences between groups at the end of the experiment (*p* < 0.05).

During the experimental period, ten plants from each of the EK-P1 to EK-P4 and P treatments were sampled every 20 days. The height and biomass of both shoots and roots were measured, as shown in **Figure 7**. After approximately 20 days, plant height in all EK-P and P groups stabilized, indicating that tall fescue reached maturity around this time. From day 20 onward, root and shoot lengths in the EK-P treatments consistently surpassed those in the P group. By day 60, both shoot/root length and biomass in the EK-P1 to EK-P4 groups exceeded those in the P group by 9.0%, 21.0%, 20.4%, and 17.6% (length), and 9.0%, 20.2%, 15.6%, and 12.8% (biomass), respectively.

**Figure 8:**
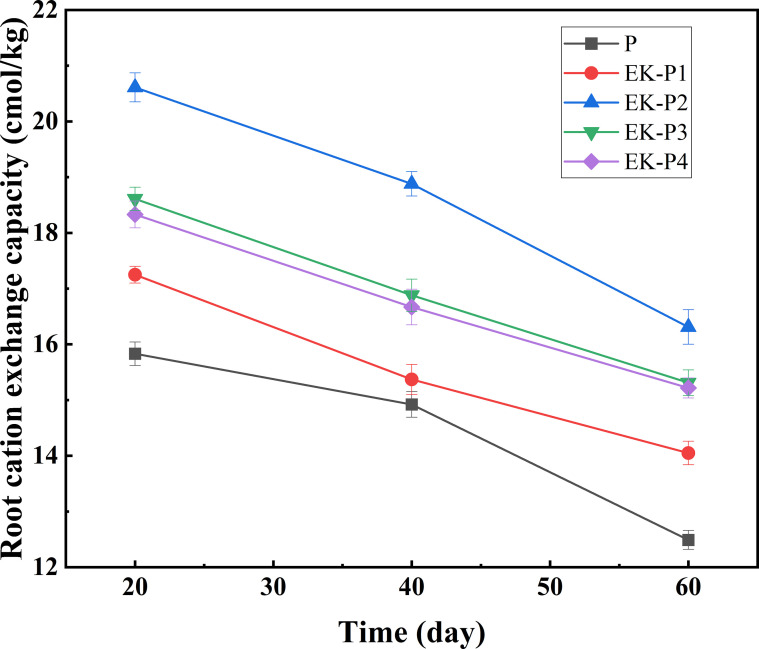
Root cation exchange capacity of tall fescue in EK-P 1-EK-P4 and P treatment (mean ± SD, n = 3).

The CEC of plant roots indicates their ability to adsorb soil cations. Through mechanisms such as mass flow, interception, and diffusion, plants attract cations to the rhizosphere, influencing their migration within the soil. As shown in **Figure 8**, root CEC decreased over time in all groups. However, in the EK-P treatments, root CEC initially increased with planting density before declining, forming a unimodal pattern. After 60 days, the CEC of tall fescue roots in the EK-P1 to EK-P4 treatments was 12.5%, 30.6%, 22.6%, and 21.8% higher, respectively, than in the P group, with EK-P2 exhibiting the highest overall CEC.

High concentrations of petroleum-contaminated soil can affect the growth of tall fescue to some extent; however, under low petroleum concentration conditions, this impact is negligible (Mottaghi et al., 2022). Moreover, all experimental control groups were conducted under the same level of petroleum contamination. By employing a controlled variable experimental design, the potential influence of petroleum on tall fescue growth was effectively eliminated as a confounding factor (Zand et al., 2010) These results indicate that the application of a periodically reversed electric field was associated with improved tall fescue growth in our system. A plausible explanation is enhanced redistribution of water and ions driven by electromigration and electro-osmosis under polarity reversal, which could increase root access to resources (Zhou et al., 2022). In the future research, this approach can be deeply analyzed and proved by measuring electroosmotic flow, nutrient ion flux or rhizosphere nutrient availability. As a result, plants are better equipped to absorb water and nutrients, accelerating growth, biomass accumulation, and ultimately increasing root CEC (Chandrasekaran, 2022). However, excessive planting density (EK-P3 and EK-P4) led to reduced plant height and biomass compared to EK-P2, likely due to intraspecific competition under high-density conditions, which limits access to light, nutrients, and space, suppressing plant growth and root development, and negatively affecting CEC (Vysotskaya et al., 2023). Conversely, EK-P1 showed weaker plant performance and limited impact on soil ion retention and structural protection, likely due to insufficient root density at low planting densities, which weakens the interactions between plant roots and soil. Since electrokinetic-plant synergy occurs primarily at the root-soil interface, sparse root systems limit this interaction, reducing the electric field’s effectiveness in promoting plant growth and resulting in relatively low root CEC in EK-P1. In contrast, EK-P2 produced optimal results, supporting robust plant growth and a well-developed root system, thereby enhancing the synergy between electrokinetic processes and plant activity. This synergy contributed to higher root CEC and positively impacted overall remediation performance.

In summary, root-soil interactions play a pivotal role in driving the synergy between electrokinetic and phytoremediation processes. While increased planting density improves root density and enhances this synergy, overly dense planting can suppress growth and lead to excessive nutrient uptake, ultimately weakening the electrokinetic-phytoremediation effect. To visually synthesize the density-dependent synergy and the links between electrokinetic forcing and plant-mediated processes, we provide a conceptual model in **Supplementary Figure 1**. In this schematic, solid arrows indicate relationships directly supported by measurements in this study, whereas dashed arrows denote plausible mechanisms inferred from the observed trends and prior literature.

## 4 Conclusion

Petroleum-contaminated soils remain an environmental concern, and electrokinetic-assisted phytoremediation offers a low-impact remediation option whose performance depends on system design. In this study, planting density exerted a non-linear influence on electrokinetic–phytoremediation performance. TPH removal and the synergistic enhancement (SE) increased from low density to an intermediate density and then slightly decreased at excessive density. Across the tested range (0.5–3 plants/cm^2^), the intermediate density of 1 plant/cm^2^ yielded the highest mean TPH removal and the largest SE, while the difference between 1 and 2 plants/cm^2^ was small. Mechanistically, our measured trends in current, pH/ions, and zeta potential/EDL suggest that intermediate densities best balance (i) maintaining soil ionic conductivity and electrical current, (ii) moderating pH and limiting colloid destabilization, and (iii) sustaining plant growth and root cation-exchange capacity. Because some mechanistic processes (e.g., exudate chemistry, precipitation phases, and physical-structure changes) were not directly quantified, we interpret these mechanisms as plausible contributors rather than proven causal pathways. These findings highlight planting density as an actionable parameter for optimizing EK-assisted phytoremediation of petroleum-contaminated soils.

For clarity, the experimental densities correspond to 5,000–30,000 plants/m^2^, which are higher than typical agronomic stand densities and reflect the confined geometry and short-term, controlled microcosm conditions used to achieve sufficient root–soil contact within 60 days. For field implementation, planting density should be translated into practical seeding rates and target root-length density for the specific plant species and soil type. Accordingly, pilot-scale trials are needed to validate the density–performance relationship under heterogeneous field conditions and to jointly optimize other scalable parameters (e.g., electrode spacing, voltage gradient, polarity-reversal frequency, and irrigation management). Future studies should also incorporate compositional hydrocarbon analyses and microbial measurements to better resolve degradation pathways and to strengthen causal interpretation of rhizosphere-driven mechanisms.

## Data Availability

The original contributions presented in the study are included in the article/**Supplementary Material**. Further inquiries can be directed to the corresponding author.
