# Supplementary material for: Optimizing planting density for enhancing the synergistic effect of electrokinetic-phytoremediation in petroleum-contaminated soils
Source: Front Plant Sci. 2026 Apr 13;17:1810883. doi: 10.3389/fpls.2026.1810883 (PMC13111288; doi:10.3389/fpls.2026.1810883)
Supplement: Supplementary file 1 [file DataSheet1.docx]

Supplementary Material

**Table S1** Initial characteristics of the soil used in the experiment

| **Soil properties** | | **value** |
| --- | --- | --- |
| pH | | 7.7 |
| Organic matter (g·kg^-1^) | | 19.8 |
| Conductivity (mS ·cm^-1^) | | 0.248 |
| Soil Texture (mm, %) | < 0.002 | 24.1 |
|  | 0.002- 0.02 | 60.2 |
|  | 0.02- 2 | 15.7 |
| Available phosphorus (mg·kg^-1^) | | 23.5 |
| Soil field capacity（%） | | 30.5 |
| Water soluble ions (mg·kg^-1^) | Ca^2+^ | 115.03 |
|  | Mg^2+^ | 23.01 |
|  | Na^+^ | 38.87 |
|  | K^+^ | 9.73 |

**Table S2** Overview of experimental treatments

|  | Treatment | Planting density |
| --- | --- | --- |
| 1 | EK | - |
| 2 | EK-P1 | 0.5plants/cm^2^ |
| 3 | EK-P2 | 1 plants/cm^2^ |
| 4 | EK-P3 | 2 plants/cm^2^ |
| 5 | EK-P4 | 3 plants/cm^2^ |
| 6 | P | 1 plants/cm^2^ |
| 7 | CK | - |

**Table S****3** Correlation coefficient matrices of TPH removal (%) and zeta potential, cation concentration, root length, plant height

|  | Zeta potential | Cation concentration | Root length | Plant height | TPH removal (%) |
| --- | --- | --- | --- | --- | --- |
| Zeta potential | 1 |  |  |  |  |
| Cation concentration | 0.525 | 1 |  |  |  |
| Root length | -0.999^**^ | -0.559 | 1 |  |  |
| Plant height | -0.999^**^ | -0.558 | 1 | 1 |  |
| TPH removal (%) | -0.843^*^ | -0.900^*^ | 0.864^*^ | 0.864^*^ | 1 |

Note：^*^ Correlation was significant at P < 0.05.


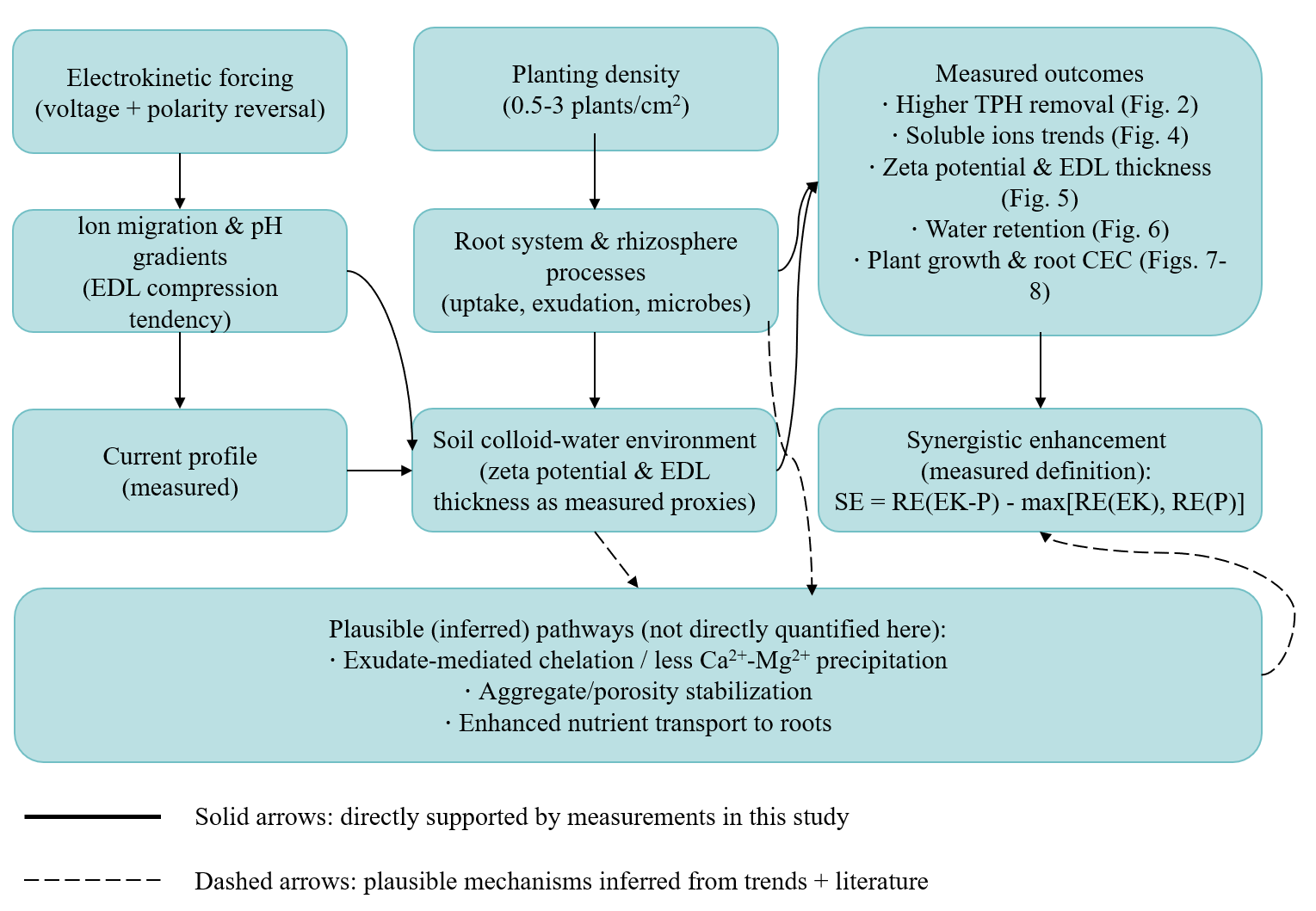


**Figure S1** Conceptual model summarizing the synergistic mechanisms of electrokinetic-phytoremediation and how planting density modulates them. Solid arrows denote relationships directly supported by measurements in this study (TPH removal, electric current profile, soluble ions, zeta potential/EDL thickness, soil water retention, plant growth, and root CEC). Dashed arrows denote plausible mechanisms inferred from observed trends and prior literature (e.g., exudate-mediated chelation/precipitation control, aggregate/porosity stabilization, and enhanced nutrient transport to roots).
